# Friction Stir Welding Optimization of 3D-Printed Acrylonitrile Butadiene Styrene in Hybrid Additive Manufacturing

**DOI:** 10.3390/polym14122474

**Published:** 2022-06-17

**Authors:** Nectarios Vidakis, Markos Petousis, Apostolos Korlos, Nikolaos Mountakis, John D. Kechagias

**Affiliations:** 1 Mechanical Engineering Department, Hellenic Mediterranean University, Estavromenos, 71410 Heraklion, Greece; markospetousis@hmu.gr (M.P.); mountakis@hmu.gr (N.M.); 2 Department of Industrial Engineering and Management, International Hellenic University, 14th km Thessaloniki-N. Moudania, Thermi, 57001 Thessaloniki, Greece; apkorlos@ihu.gr; 3 Department of Forestry Wood Science and Design, University of Thessaly, 43100 Karditsa, Greece; jkechag@uth.gr

**Keywords:** friction stir welding (FSW), acrylonitrile butadiene styrene (ABS), material extrusion (MEX), hybrid additive manufacturing, 3D printing

## Abstract

The feasibility of joining material extrusion (MEX) 3D-printed acrylonitrile butadiene styrene (ABS) plates with the friction stir welding (FSW) process was investigated herein as a promising topic of hybrid additive manufacturing (HAM). The influence of three process parameters on the mechanical strength of the joints was thoroughly examined and analyzed with a full factorial experimental design and statistical modeling. Hereto, the welding tool pin geometry, travel speed, and rotational speed were investigated. The joint’s efficiency and quality are evaluated through tensile tests and morphological characterization. More specifically, specimens’ areas of particular interest were investigated with stereoscopic, optical, and scanning electron microscopy. Throughout the FSW experimental course, the welding temperature was monitored to evaluate the state of the ABS material during the process. The majority of the welded specimens exhibited increased mechanical strength compared with the respective ones of non-welded 3D printed specimens of the same geometry. Statistical modeling proved that all processing parameters were significant. The feasibility of the FSW process in 3D printed ABS workpieces was confirmed, making the FSW a cost-effective process for joining 3D printing parts, further expanding the industrial merit of the approach.

## 1. Introduction

FSW is an autogenous continuous solid-state welding method performed with a rotating welding tool that heats the welding parts through friction. It was developed primarily for metals that are difficult to weld [1]. Aluminum sheets are thoroughly studied in the literature for different grades, such as AA1050 [2,3], AA7075 [4,5,6], and AA6013 [7]. Research in FSW for polymers is still very poor [8]; still, specific parameters have been investigated in the literature [9]. The aim was to find the optimal conditions [10] for the FSW process. FSW for 3D printed parts, according to the authors’ knowledge, is still marginal. Different polymers and their composites have been studied [11,12,13]. The parameters for welding polyethylene through the FSW process have been reported in the literature [14,15], and research has also been carried out on the fracture of the welded parts [16]. The performance of Poly(methyl methacrylate) (PMMA) sheets welded with FSW has been investigated, too [17]. Nylon 6 has been processed with FSW on a heat-assisted experimental setup [18]. The performance of its fiber-reinforced composites has also been studied, aiming to improve the process [19]. Joining different polymers with FSW has been investigated for various polymers, for example, PMMA welded with acrylonitrile butadiene styrene (ABS) [20]. Additionally, polymers have been welded with other materials as well, such as wood–plastic composites [21], aluminum [22], or metals [23,24], with the FSW process. The welding tools’ performance has also been investigated, as their effect on the result of the process is critical [25].

Statistical modeling tools have been introduced for the analysis and optimization of material extrusion (MEX) 3D printing parameters for materials, such as Polyamides [26] and Thermoplastic Polyurethane (TPU) [27]. Additionally, such modeling tools have been used for the optimization of the surface quality of 3D printed parts of different materials [28], and in hybrid additive manufacturing (HAM), for laser cutting of polymers 3D printed with MEX, such as polylactic acid (PLA) [29], and polyethylene terephthalate glycol (PETG) [30]. These modeling tools revealed the critical parameters in each case, leading to useful conclusions for the improvement of the results of these processes. In FSW, arithmetical modeling tools, such as Finite Element Analysis (FEA), have been applied [31], with research also focusing on the simulation and the investigation of volumetric defects during the FSW process [32]. Various computational or statistical models have also been introduced for the study of process performance. The research concluded that the process parameters have a substantial effect on the welding grade [33], with the rotational tool being the dominant parameter in the process [34].

**Figure 1:**
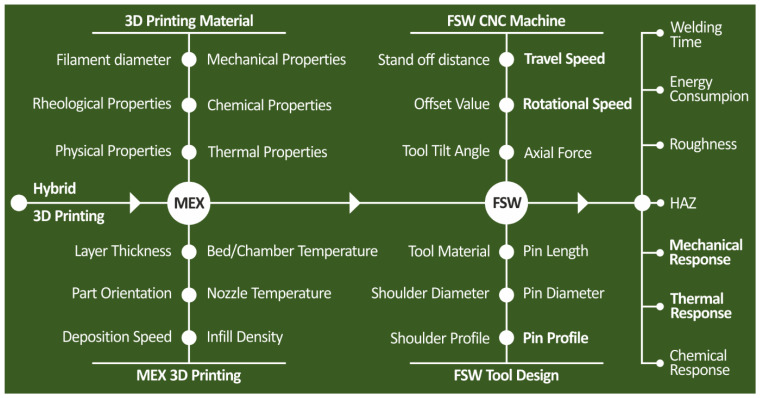
MEX 3D printing and FSW process parameters and the welding performance features.

The key parameters of the hybrid MEX/FSW process are depicted in Figure 1 below through a cause-and-effect diagram. The produced weld quality and the overall performance of the joints are affected by the 3D printing parameters used to fabricate the workpieces and the FSW parameters applied to joint them.

Herein, the feasibility of joining 3D printed ABS parts was investigated. Although ABS has been thoroughly investigated in MEX 3D printing [35,36,37,38] and hybrid AM, combining 3D printing with laser cutting [39], no study is yet available on the feasibility and the effect of FSW on MEX 3D printed ABS. A deeper insight into the main difference between FSW on polymer-based parts produced by mainstream processes (extrusion, rolling, etc.) and FSW on polymer-based parts produced by 3D printing was presented in this work. Statistical tools, such as the design of experiment (DOE) and quadratic regression models (QRM) were employed to specify the result of the FSW processing variables on the weld result. No similar study is yet available in the literature for the FSW of MEX parts. Although joining polymeric parts with FSW has been presented in the literature, welding MEX polymeric parts is a challenging process, due to the parts’ built structure, their porosity, and the anisotropy in their behavior. This was achieved herein for the first time and a first attempt to assess the effect of the FSW variables on the weld result was made. Joining 3D polymeric parts has great potential, since parts that cannot be built with conventional manufacturing methods, due to their geometry or other factors, can be 3D printed and welded with the process. Additionally, large-scale parts that cannot be 3D printed as one part with polymeric materials, due to their size, can also be 3D printed and joined with FSW. Such manufacturing capabilities can be useful in different industries, such as packaging, shipbuilding, aerospace, automotive, and electronics [9,40,41,42,43]. Statistical modeling tools also revealed the dominant parameters in the process, among the ones studied. The parameters investigated were the welding tool pin geometry (PPA, PPB), the tool rotational speed (RS; 3 values), and the travel speed (TS; 3 values), while all the other parameters (FSW and MEX) were kept constant, for comparison purposes. Preliminary calibration experiments were implemented to verify the test setup’s operation and determine the parameter ranges for the full factorial experimental course that followed. The weld results were evaluated with tensile testing to determine the mechanical response of the welded specimens for all the different cases studied. The morphology of the produced weld was evaluated with images taken with a stereoscope, optical, and SEM microscope. The temperature developed during the welding experiments was also monitored and recorded to verify whether the solid state on the specimens, which is required for the FSW process, was maintained. The effect of this parameter on the weld result was also evaluated. Overall, all processing parameters affected the mechanical performance of the welded specimens, with travel speed dominating in the tensile modulus (E) and welding temperature (WT), while rotational speed (RS) dominated in the tensile strength (sB) of the specimens. The mechanical performance of the welded specimens was generally better than the non-welded specimens 3D printed with the same conditions and geometry, owing to the reduced porosity of the welded area the FSW process induces to the welded samples. This agrees with similar findings in the literature [44].

## 2. Materials and Methods

**Figure 2:**
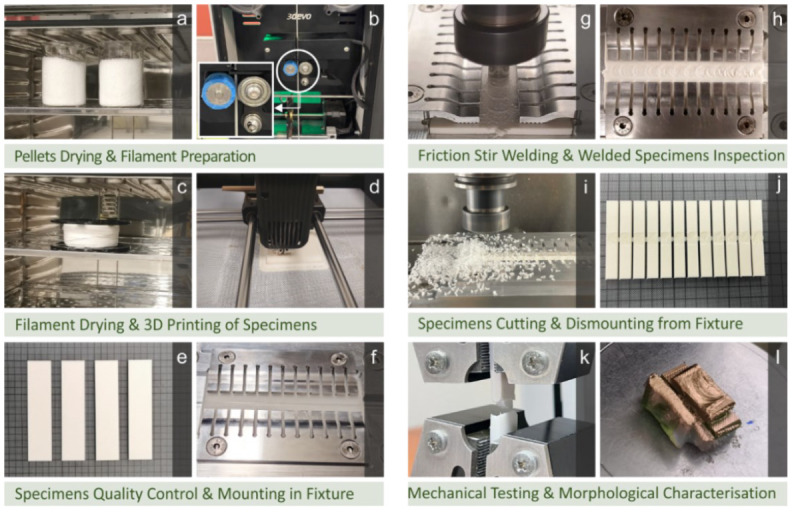
(**a**) Raw material drying, (**b**) filament extrusion, (**c**) filament drying, (**d**) workpieces 3D printing, (**e**) workpieces for the FSW process, (**f**) FSW experimental setup, (**g**) FSW experiments, (**h**) FSW process weld seam, (**i**) workpiece cutting in the milling machine, (**j**) workpieces dismounted from the fixture, (**k**) tensile testing, and (**l**) SEM morphological characterization.

In this work, the ability to join 3D-printed parts made of ABS with the FSW process was experimentally investigated. Figure 2 presents the flow chart of the current research.

ABS was selected as it is the second-most popular material in MEX 3D printing [45]. For the preparation of the experiments’ workpieces, industrial-grade Terluran Hi-10 ABS in powder form was procured from INEOS Styrolution (INEOS AG, Frankfurt, Germany). The raw material was initially dried at 70 °C for 24 h and then used to fabricate filament, with a thermomechanical extrusion process, on a single-screw extruder (3D Evo Composer; 1.75 mm filament diameter). The filament was used to manufacture the workpieces for the FSW process on a Zortrax M300 Dual 3D printer (Zortrax, Olsztyn, Poland). The 3D printing settings used are shown in Table 1.

**Figure 3:**
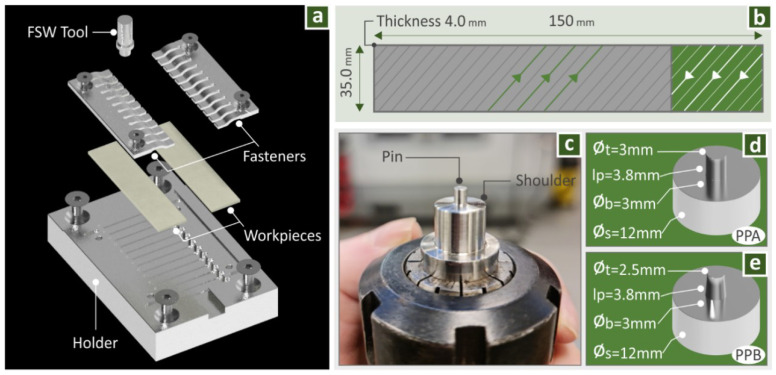
(**a**) Featuring the two workpieces used in each round, the welding tool, and the fixture assembly, (**b**) 3D printed workpieces dimensions and infill pattern, (**c**) welding tool, (**d**) welding tool PPA (cylindrical) dimensions, and (**e**) welding tool PPB (frustum) dimensions.

The geometry and the infill pattern on the workpieces are shown in Figure 3b. Their dimensions were selected to be suitable for the fixture used in the work, which is presented in Figure 3a. In this Figure the assembly of the experimental setup is presented, featuring the operating principle of the conducted FSW experiments. In each experiment, a straight-line joint was created between two identical workpieces, in their contact surface along their length direction. In each FSW round, with the fixture and the workpieces used, twelve specimens with 10 mm width for tensile testing were produced. These were automatically cut in the milling machine after the welding process. It was chosen to have three different welding conditions in each round, so the travel speed was changed three times in equal lengths, producing three sets of four specimens, welded with identical FSW parameters.

For the FSW process, two different welding tools were used to evaluate the effect of their geometry on the weld result. Their dimensions were selected based on generic information from the literature (same shoulder, cylindrical, and frustum pin respectively) and are shown in Figure 3d (Profile Pin A, PPA) and Figure 3f respectively (Profile Pin B, PPB) [46]. The tools were manufactured in a Haas SL20 lathe (Haas automation Inc., Oxnard, CA, USA), from AISI304L stainless steel bars, and they were post-processed with st3000 sandpaper, for polishing of their surfaces, to achieve average surface roughness Ra 0.05 μm.

FSW experiments were conducted on a Haas TM-1P CNC machine (Haas automation Inc., Oxnard, CA, USA). The G-code for the process was programmed directly in the machines’ MCU. During the FSW process, the temperature was monitored and recorded with a Flir One Pro thermal imaging camera.

Additionally, TGA and DSC were utilized to examine the thermal properties of the ABS material of the study, for evaluating its state during the experiments. TGA measurements were carried out using a Perkin Elmer Diamond TGA apparatus (Perkin Elmer Corp., Waltham, MA, USA) (heating cycle: 30–550 °C, heating step: 10 °C/min). The DSC measurements were performed using a TA Instruments DSC 25 apparatus (Waters Corp, New Castle, DE, USA) (heating cycle: 25–220–25 °C, heating step: 15 °C/min).

Initial trials for the proof of concept and the calibration of the procedure were conducted with the experimental setup described above. Workpieces with two different colors were used in each test in an attempt to show the mixing of the materials during the FSW process. The FSW parameters at this stage were selected from the literature [47,48]. At this point, the process feasibility was confirmed. In these prescreening experiments before the full factorial experimental design selection (parameters and their levels selection), the aim was to document the weldability of the layered workpieces and determine the range of the control parameters that produce acceptable results (seam quality, thickness decrease, etc.). This approach was decided mainly due to the anisotropic properties of the welded plates, which makes it difficult to make inferences and hypotheses for the FSW 3D printed materials. So, after the experimental results and having appropriate welding efficiency, the authors concluded that the optimization followed here was reasonable.

So, after this preliminary extensive work and literature review, the designated variables and their values were (see Table 1): (i) the tool pin profile (PPA, and PPB), (ii) the tool RS (600, 1000, 1400 rpm), and (iii) the tool TS (3, 6, 9 mm/min). The measured output parameters were the following: (i) the tensile strength (sB: MPa) on welded area, (ii) the tensile modulus of elasticity (E: MPa), and (iii) the maximum welding temperature (WT: °C). Based on this parameter design, the full factorial experimental approach was decided with three repetitions for each discrete combination resulting in fifty-four experiments (2 × 3 × 3 × 3 = 54). Regarding the WT, it should be noted that although there is sufficient analytical and numerical work in the literature for the temperature course throughout the steady-state (quasi-static approaches) part of the FSW process for metals and/or metal alloys, the corresponding works for polymeric-based parts are rather limited (mainly by Derazkola et al., for PMMA [49] and PC [50] sheets welding), which extend forward existing sophisticated and well-established FEA models for metals and metallic alloys. The basic assumption of these models is that the workpieces (specimens to be welded) are bulky in behavior, i.e., they are made of homogeneous and isotropic materials (e.g., the intrinsic material properties, such as physical properties, mechanical properties, thermal conductivity, and other thermal properties, viscosity, etc., are identical to all three dimensions). This assumption is well based for metals and metal alloys and quite acceptable for polymer-based materials if they are “fully solid” and non-layered (or without any internal macro-pattern). In the case of MEX 3D printed parts, such as the ones in our work, neither homogeneity nor isotropy occurs [51]. MEX 3D printing is well known for its flexibility to control several building parameters, such as layer height, strands’ width, infill density, raster pattern, etc., which all imply high inhomogeneity and anisotropy to the outcome, in a stochastic manner. On the other hand, the porosity (even for 100% infill density), the surface roughness, and the dimensional inaccuracy are unavoidable, especially when compared with other fabrication processes [28]. Therefore, the adoption of the aforementioned analytical and/or numerical thermo-mechanical models will not produce reliable or somehow acceptable results. Even the differences in quality of commercial filaments induce significant variations in “identical” experiments with different filaments of the same materials, produced by different vendors. The inhomogeneity and anisotropy of the 3D printed samples are more than evident in several micrographs and SEM photos throughout the figures presented below in this work.

The output values were utilized to construct the main effect plots (MEP) of the variables against the three outputs and then decompose the interactions between parameters vs. WT, sB, and E. Finally, three QRMs were adopted to predict the output values according to the input values. The linear, cross, and quadratic products of the QRMs were evaluated by Analysis of variance (ANOVA) analysis to characterize their predicting accuracy by the R-square indexes.

After the completion of the FSW process, the welded workpieces were evaluated for their mechanical performance in tensile testing and their morphological characteristics. Tensile trials were carried out in an Imada MX2, at room temperature with an elongation speed of 10 mm/min. A not-welded 3D-printed ABS specimen was also tested for comparison purposes. In this way, both the performance of the weld and the weld parameters could be compared and evaluated. For the morphological characteristics, a stereoscope (KERN OZR5, KERN & SOHN GmbH, Balingen, Germany), an optical microscope (Kern OKO 1, KERN & SOHN GmbH, Balingen, Germany), and an SEM (JEOL JSM 6362LV, Jeol Ltd., Tokyo, Japan) were employed. Images at the stereoscope and the optical microscope were captured using a KERN ODC 832 5MP camera (KERN & SOHN GmbH, Balingen, Germany). SEM images were captured on sputter-coated gold (Au) specimens in high-vacuum mode with a 20 kV acceleration voltage. Images were taken at various areas, with various magnifications in the Heat Affected Zone (HAZ) and the transitional area of the weld, to thoroughly investigate any phenomena in the regions.

## 3. Results and Discussion

**Figure 4:**
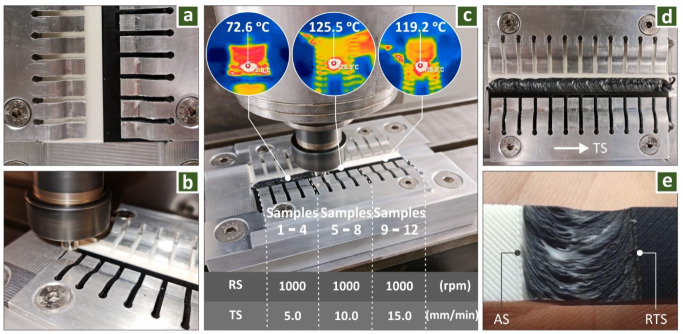
(**a**) ABS workpieces with two different colors for the proof-of-concept experiment, fixed in the fixture, (**b**) proof of concept experiment, (**c**) conditions for the FSW experiment, and monitoring of the temperatures during the FSW experiment, (**d**) weld seam produced during the calibration of the process, and (**e**) detail of the weld seam.

The proof of concept stage of the work is presented in Figure 4. In these welding tests, a black and a white 3D-printed ABS specimen (Figure 4a) were welded with the experiment setup of the work, in an attempt to visualize and inspect the mixture of the materials during the process. Figure 4b shows such an FSW experiment. Each FSW round used three different sets of weld conditions, and four specimens were created for each set of parameters, as previously stated. The parameters used in these experiments along with the temperature recorded in each region welded with the same parameters are shown in Figure 4c. The weld seam produced in one of the calibration stage experiments is presented in Figure 4d. Figure 4e shows a detail of the weld seam, in which the retreating side (RTS) and the advancing side (AS) are indicated. The width of the weld seam and the HAZ, in general, are affected by the shoulder diameter and in this case were in good agreement, as is shown in the image.

**Figure 5:**
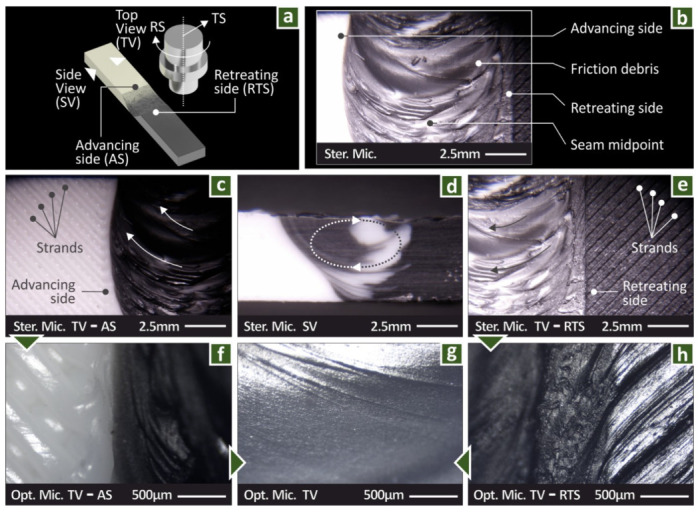
(**a**) The specimens’ different areas, (**b**) HAZ view, (**c**) advancing side view, (**d**) cross-section of the welding zone, (**e**) view of the retreating side, (**f**) advancing side view, (**g**) welding zone view, (**h**) retreating side view.

Figure 5 shows typical stereoscopic and optical microscopy images from the workpieces welded during the proof of concept stage. Figure 5a indicates the different areas examined in the specimens during the inspection with microscopy. The AS and RTS sides in the diagram are defined by the TS direction and the RS orientation, respectively (Clockwise—CW, Counterclockwise—CCW). The AS is on the side of the white workpiece in this Figure, whereas the RTS is on the side of the black workpiece. A stereoscopic image of the joint obtained from the upper side of the specimen is shown in Figure 5b. The different zones are visible in the image, along with the characteristic onion ring pattern of the FSW process. Figure 5c,f show in different magnifications the AS. In the lower-magnification image (Figure 5c) the strands outside the HAZ are visible. Figure 5d shows the HAZ from the side. The circular movement of the material during the mixing process is visible in the image, with the material of the RTS (black material) moving to the side and the material from the AS (white) moving towards the center of the HAZ. Figure 5g shows a detail of the welding zone, in which the flow of the material is shown. Figure 5e,h show different magnifications the RTS. Similar observations to the AS can be made, while more rough surface patterns are presented.

**Figure 6:**
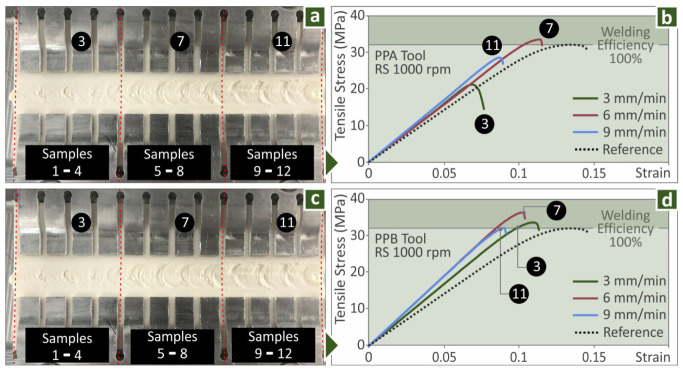
(**a**) FSW with the PPA, (**b**) stress–strain graphs of specimens with the PPA tool, (**c**) FSW with the PPB, (**d**) stress–strain graphs of specimens with the PPB tool.

After the proof of concept stage, the final FSW parameters were determined. With these parameters, a full factorial experimental design was implemented. All the combinations of the parameters were experimentally tested. Figure 6a shows the welding seam of workpieces welded with the PPA tool (cylindrical pin), while Figure 6c shows the corresponding welding seam with the PPB tool (frustum pin). As described above, after the completion of the FSW, the welded workpiece was automatically cut in the milling machine into specimens that were tested for their mechanical performance in tensile tests. Figure 6b shows typical stress–strain curves for specimens welded with various TS values with the PPA tool and Figure 6d shows the corresponding graphs for the PPB welding tool. A similar pattern was observed with the workpieces welded with 6mm/min TS exhibiting higher mechanical response. For comparison purposes, a not-welded ABS MEX 3D-printed specimen (manufactured with the same settings and the same dimensions) was tested as a reference. Specimens welded with the PPA tool showed adequate strength, but it was lower than the reference specimen. Specimens welded with the PPB tool showed similar or higher strength than the reference specimen. This verifies that the welding tool affects the result of the process and it can lead to an enhancement of the mechanical properties of the MEX parts. The reference specimen showed overall a more ductile behavior than the welded specimens.

One of the measures of the performance of a weld is the welding efficiency, which is defined as the ratio of the tensile strength of the weld and the tensile strength of the original, not welded, material [52]. In this work, in several cases, the welding efficiency was higher than 1, showing that the weld has higher strength than the original 3D-printed part.

The mechanical strength of the welded specimens can be attributed to the reduction of the porosity in the material structure in the HAZ. The process produces a more solid structure in the material in the welded area. This was also verified by the fact that most of the specimens failed outside the HAZ in the tensile tests, showing the enhanced strength of the mixed material developed in the region, due to the reduction or absence (optically observed) in some cases of porosity.

**Figure 7:**
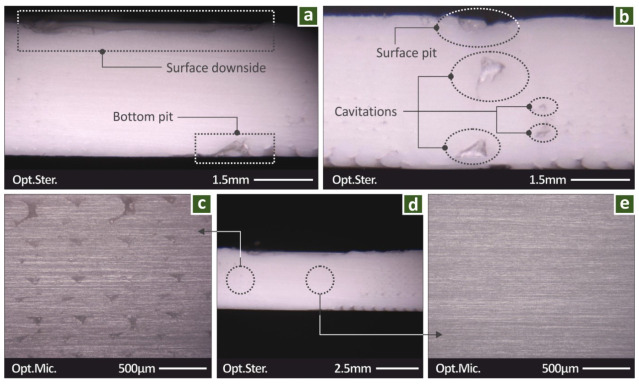
Side views of the welded specimens: (**a**,**b**) stereoscopic images of the HAZ, and HAZ limits, (**c**) view outside the welding zone, (**d**) stereoscopic image at lower magnification, and (**e**) view of the welding zone.

Figure 7 shows microscope images from the side surface of a welded specimen. A minor decrease in the height of the specimen is observed in Figure 7a, attributed to material filling the voids and thus reducing porosity in the weld area. In Figure 7a,b, defects in the specimens are present in the form of surface pits and cavitations. Surface pits can be attributed to the FSW process, while cavitations can be attributed to the cut process of the specimen in the milling machine. Figure 7d shows the regions in which Figure 7c,e were taken. Figure 7c was taken outside the HAZ and filament strands are visible from the 3D-printed structure in the specimens. Figure 7e shows a corresponding image within the HAZ and the material is solid, without any porosity. This effect of the FSW process, as mentioned above, led to the failure of the specimens outside the HAZ in most cases and the increase in the mechanical strength of the welded specimens in specific cases when compared with the not-welded ones.

**Figure 8:**
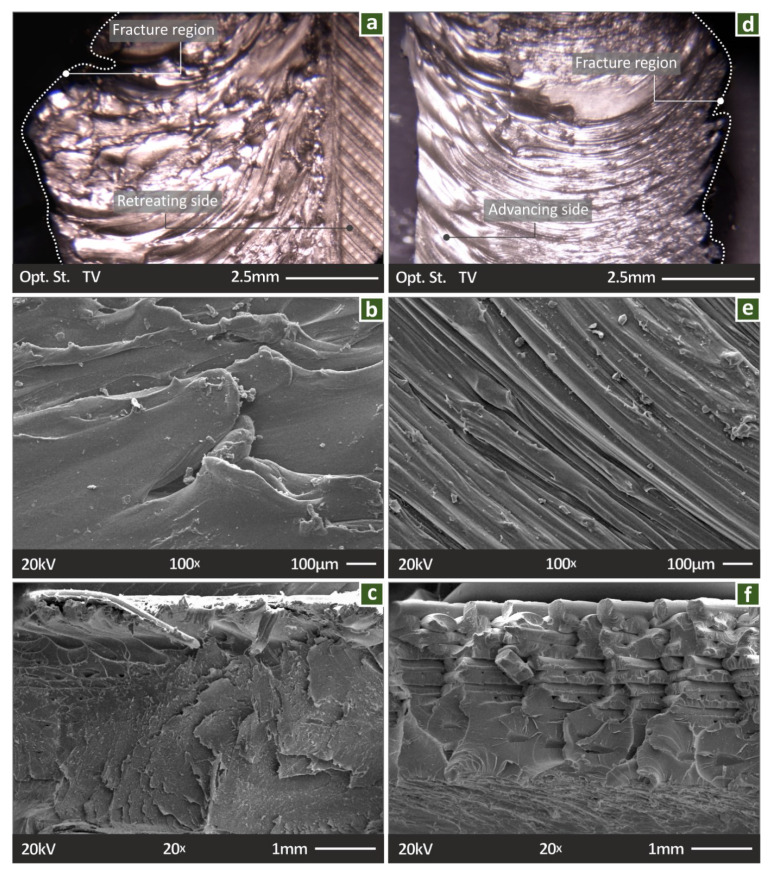
PPA tool: (**a**) top view of the seam, (**b**) SEM top view at the center of the joint, (**c**) SEM image of the fractured cross-section of the joint; PPB tool: (**d**) top view of seam, (**e**) SEM top view at the center of the joint, and (**f**) SEM image of the fractured cross-section of the joint.

Figure 8 shows stereoscopic and SEM images from the gold-sputtered tensile test specimens (one welded with the PPA tool, Figure 8a–c and one welded with the PPB tool, Figure 8d–f), after they fail in the test, to evaluate their morphology and the fracture mechanism during the tests. Figure 8a,d are stereoscopic images from the top of the specimens at the fracture area. As it is shown, both specimens did not fail in the weld area, but in the transitional area between the welded and the non-welded area. The fracture profile from the top can be evaluated in the images. The RTS (Figure 8a) and the AS (Figure 8d) are shown, respectively. Figure 8b,e show SEM images from the HAZ of the two specimens. The FSW onion rings are visible in the PPB specimen (Figure 8e), while they are not so obvious in the PPA specimen (Figure 8c), although material flow can be observed. Figure 8c,f show SEM images from the fracture surface of the specimens after the tensile tests. The specimen welded with the PPA tool showed a more brittle behavior than the specimen built with the PPB tool. Additionally, the PPA specimen failed in the transitional region of the HAZ, while the PPB specimen failed outside the weld region; hence, the 3D-printing structure is visible in the SEM image of the fracture area. Overall, specimens welded with the PPA tool showed more rough surfaces than the specimens built with the PPB tool, which show a more ductile response. These differences presented in all the corresponding images show that the welding tool affected the results of the FSW process. This was verified in the results of the mechanical tests as well, in which PPB specimens showed an overall higher mechanical response than the corresponding specimens welded with the PPA tool.

**Figure 9:**
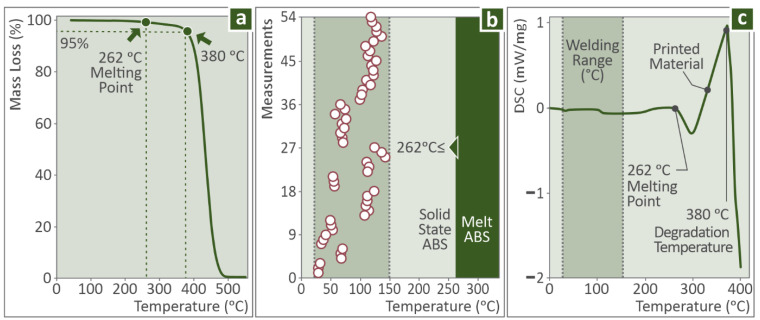
(**a**) TGA of the ABS, (**b**) highest temperature reported, and (**c**) DSC of the 3D printed material.

The thermal properties of the ABS material used in this work were investigated with TGA (Figure 9a) and DSC (Figure 9c) measurements on the material to determine whether the experiment conditions affect its thermal stability. The TGA graph verified that the filament extrusion and the 3D printing temperature used in this work did not cause any degradation of the material. The produced DSC curve revealed the melting point of the specific ABS material.

Figure 8c shows the maximum recorded temperature in each one of the fifty-four FSW experiments conducted throughout the full factorial design course. The experimentally derived temperatures were measured in this work to ensure that the FSW experiments conducted were fully in the solid-state of the workpiece without melt occurring and to be able to correlate the temperatures and the mechanical properties of the specimens versus the various control paraments (Tool geometry, Travel Speed, and Rotational Speeds) through stochastic analysis and metrics.

**Figure 10:**
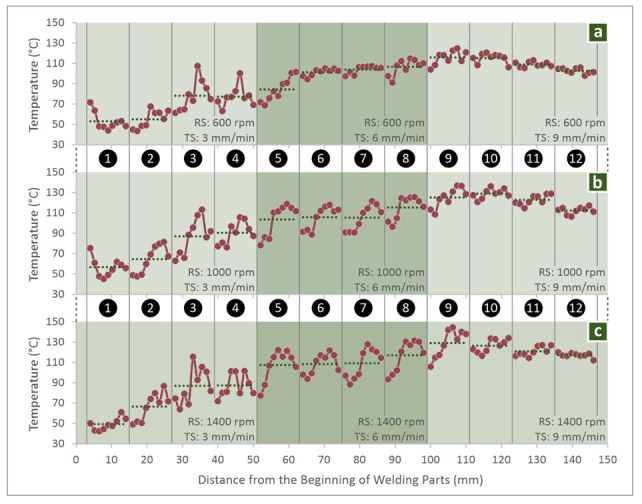
Temperature course for each specimen made with the PPB tool. Specimen welded with 3, 6 and 9 mm/min TS and (**a**) 600 rpm, (**b**) 1000 rpm, (**c**) 1400 rpm.

As it is shown in Figure 9b, the maximum temperature recorded among all the experiments was about 150 °C, which is still much lower than the material’s melting point (262 °C), as was determined in the DSC measurement. So, this verifies that the ABS was in a solid-state during the welding experiments, which agrees with the process specifications and also verifies the reliability of the produced results during the experiments. Figure 10 presents the temperature course from the beginning to the end of the weld seam during the welding process for specimens welded with the PPB tool, for every combination of the examined TS and RS. The variation of the temperature measurements (nine measurements per specimen) proves the strong stochastic nature of the temperature parameter, owing mainly to the inhomogeneity and anisotropy of the 3D printed samples, which is evident in the micrographs and SEM the photos captured in this work. Hereto, the further modeling assumption, i.e., that the FSW process at its steady-state could be approached with a quasi-static analytical or FEA analysis is, rather, a long shot that does not seem to fit in the case of 3D printed parts.

As explained in the parameter design section, the variable factors consisted of one categorical type (Tool: PPA and PPB), and two parameters with continuous values (RS and TS). With this parameter design, the full factorial design yielded eighteen combinations resulting in fifty-four final experiments after three repetitions (see Table 2). Note here that the variability of the sB(MPa) and E(MPa) values showed high variability, i.e., the maximum-minus-minimum values of E and sB were about the same as their mean values. Therefore multi-parameter optimization is vital to increase the process efficiency.

**Figure 11:**
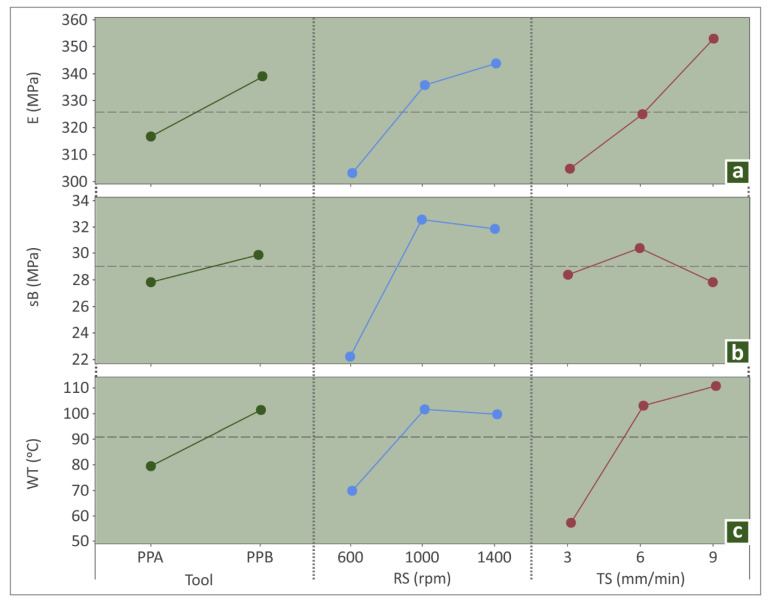
MEP plots: (**a**) E, (**b**) sB, and (**c**) WT.

MEP plots, which graphically show the effects of each input parameter level (Tool, RS, and TS) on output (E, sB and WT), can be constructed by the results presented in Table 2. For example, the MEP plots (Figure 11) reveal the following:

- Tool PPB (frustum pin) yielded better E and sB and higher temperatures (WT). This can be because the conical surface of PPB is larger than the cylindrical of PPA (see Figure 3d,e). Therefore, the side contact surface with the plasticized material was bigger and transferred a higher mass between the tool’s leading and trailing, resulting in better mixing and homogenization. Moreover, the heat produced by the contact frustum pin surface was higher, too, resulting in higher WTs in the welded area. It will be interesting to see these results in future work utilizing the Finite Element Method (FEM) and validate these observations.
- The rotational speed (RS) increases all outputs, i.e., the tensile strength, the modulus of elasticity, and the welding temperatures highly from 600 to 1000 rpm and then from 1000 to 1400 rpm, yielding higher values for the modulus of elasticity and about the same for ultimate tensile strength and welding temperature. The mixing and the homogenization of the specimens’ material on the welding seam area are caused by the tool’s rotational speed. The two welded materials shaped a lattice structure, which is highly affected by the rotational speed. More, the explanation of these observations is connected with the thermal dependence of the wear coefficient of the pin contact surface. An elevated-value frictional heat was induced and transmitted to the material during the FSW process, resulting in higher welding temperatures and improved sB and E values.
- Finally, travel speed increased from 3 to 6 mm/min and resulted in a weld with a better mechanical response (tensile strength and modulus of elasticity) and higher welding temperatures. Then, higher than 6 mm/min TS resulted in lower sB, higher E, and about the same negligible higher WT. It seems that between 3 and 9 mm/min, the TS influenced the effects of the RS in E and WT positively, while for the sB, the effect was initially positive and then negative. The friction coefficient between the tool surface and the weld material is probably the reason for the observed results. The increase of friction coefficient resulted in a higher tangential force exerted on the ABS and consequently caused the pouring of the melt material out of the welding area.

**Figure 12:**
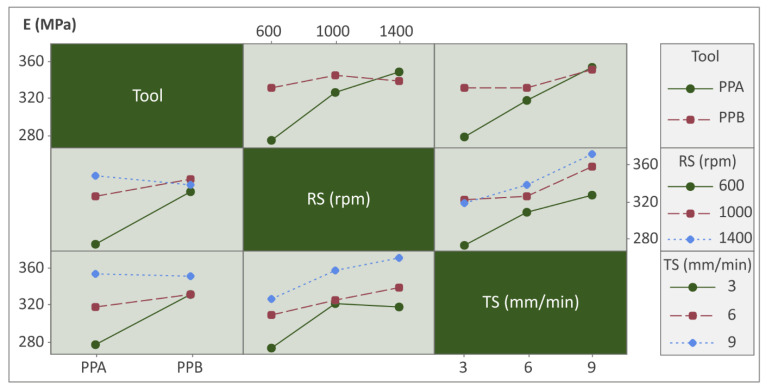
Interaction chart: Tool, RS, and TS vs. E.

**Figure 13:**
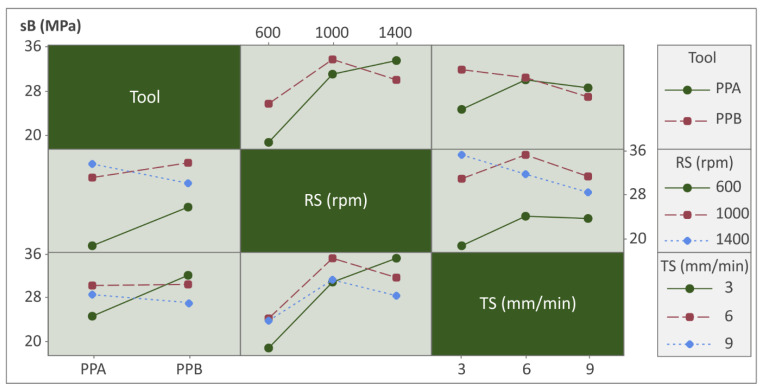
Interaction chart: Tool, RS, and TS vs. sB.

**Figure 14:**
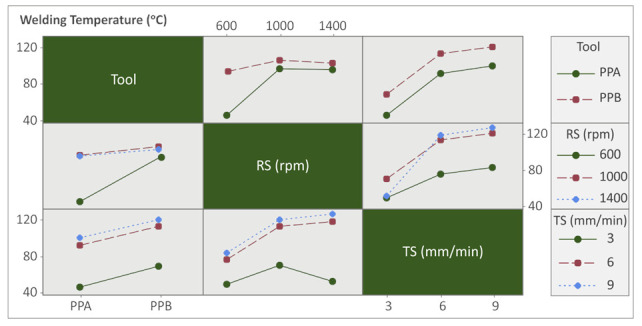
Interaction chart: Tool, RS, and TS vs. WT.

The interaction plots were adopted to see inside the processing conditions and how each parameter interacts with each other (see Figure 12, Figure 13 and Figure 14).

The trend lines between rotational speed and travel speed versus modulus of elasticity increased smoothly without changing their directions. Furthermore, the same trends were observed for the rotational speed and Tool and the travel speed and Tool versus modulus of elasticity (Figure 12), indicating that the linear models with cross products or the quadratic regression models (QRM) will be appropriate for modeling the E versus Tool, rotational speed, and travel speed variable parameters.

Figure 13 shows interactions of all input parameters versus Tensile strength (sB/MPA). In this case, the trend lines were slightly more complex, leading in some cases to a smooth change of direction. So, non-linear models such as quadratic regression models are proposed in this case.

Finally, interaction trend lines between all input parameters versus weld temperatures were in similar directions with synergistic interactions, meaning that linear or quadratic regression models will be appropriate for fitting the observed experimental data (see Figure 14).

**Figure 15:**
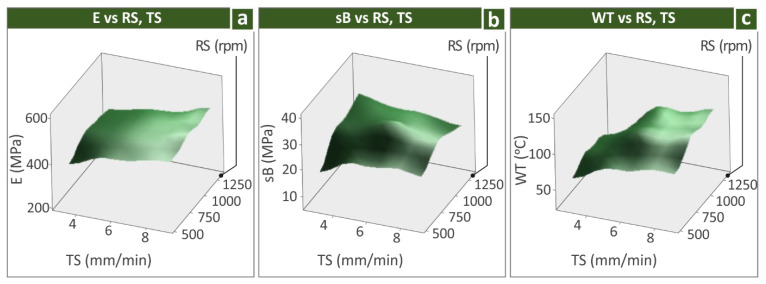
Surfaces of processing parameters vs.: (**a**) E, (**b**) sB, and (**c**) WT.

Bellow the experimental data for modulus of elasticity (E/MPa), ultimate tensile strength (sB/MPa), and weld temperature (WT, °C) outputs versus rotational speed and travel speed are shown with surface plots (see Figure 15).

After the above analysis utilizing descriptive statistical tools (MEP plots, interaction charts, and surfaces plots), quadratic regression models were adopted for all outputs in the following form:

(1) $$Y_{k}=b_{0,k}+\overset{n}{\underset{i=1}{\sum }}b_{i,k}x_{i}+\overset{}{\underset{i<j}{\sum }}b_{ij,k}x_{i}x_{j}+\overset{n}{\underset{i=1}{\sum }}b_{ii,k}x_{i}^{2}\pm e_{k}\;$$

where *Y_k_* is the output (*Y*_1_, *Y*_2_, *Y*_3_: E/MPa, sB/MPa, and WT/°C), *b* is the constant values, *x*_i_ is the input parameters (*x*_1_, *x*_2_, *x*_3_: Tool, RS, and TS), and *e* is the process error.

The analysis of variances (ANOVA) for the three quadratic regression models yielded F coefficients (Fisher values) greater than 28 and *p*-values lower than 0.05 for all outputs: E/MPa, sB/MPa, and WT/°C (see Table 3, Table 4 and Table 5).

The very high R-square indexes values for all outputs strongly indicated that these three QRMs give good predictions and can be used for processing optimization.

## 4. Conclusions

The feasibility of welding 3D printed ABS parts with FSW was confirmed in this study. The effects of three different parameters of the process were also studied with statistical modeling tools, highlighting travel speed (TS, mm/s) and rotational speed (RS, rpm) as significant processing parameters affecting the mechanical performance of the welded workpieces. A full factorial experimental design was carried out for the welding tools RS and TS, while two different welding tool geometries were tested for their effect in the process. The mechanical performance of the welded specimens was evaluated with tensile testing. The effects of the three studied parameters on the sB, the E, and the WT were evaluated. It was found that the FSW in 3D-printed ABS parts is not only feasible, but it leads to an enhancement of the mechanical performance of the welded parts when compared with the respective 3D-printed not-welded ones. The tool geometry, RS, and TS processing parameters significantly influenced the quality of mixing and homogeneity for the welded material. For example, frustum tools and higher RSs improved E, sB, and WT, while higher TSs resulted in higher tangential forces and higher WT and E values and about the same sB.

To further investigate the weld results, images were taken and evaluated. The solid-state of the ABS material during the FSW was verified by monitoring the developed temperature throughout the process in all conducted experiments.

The results of the work significantly enhance the industrial merit of 3D printing. The use of the technology can be expanded in areas where it is not possible to be exploited nowadays, such as the manufacturing of large-scale parts. In future work, additional FSW parameters can be tested and studied, and parameters can be tested at different value ranges.

## Figures and Tables

**Table 1:** MEX-FSW parameters.

	Abbr.	Units	Values
3D-printing parameters (constant)			
Nozzle Diameter:	ND	Mm	0.4
Layer Thickness:	LT	Mm	0.20
Infill Density	ID	%	100 (Solid)
Printing Temperature	PT	°C	275
Platform Temperature	PT	°C	90
FSW parameters (variable)			
Rotational Speed	RS	Rpm	600, 1000, 1400
Travel Speed	TS	mm/min	3, 6, 9
Pin Profile	PP_	A: cylinder, B: taper	PPA, PPB

**Table 2:** Experimental measurements.

	Input			Output				
No	Tool	RS (rpm)	TS (mm/min)	WT (°C)	sB (MPa)	E (MPa)	sB/sB ^1^ (%)	E/E ^2^ (%)
1	1	600	3	29.2	9.7	194.6	30.2%	67.1%
2	1	600	3	29.0	10.5	207.4	32.5%	71.6%
3	1	600	3	31.7	13.3	243.4	41.3%	84.0%
4	1	600	6	53.2	23.1	295.5	71.3%	102.0%
5	1	600	6	50.8	22.5	288.7	69.7%	99.6%
6	1	600	6	49.2	21.3	282.3	65.9%	97.4%
7	1	600	9	56.3	23.6	323.7	73.0%	111.7%
8	1	600	9	55.5	22.4	337.4	69.3%	116.4%
9	1	600	9	53.8	21.6	297.8	66.7%	102.8%
10	1	1000	3	68.1	26.9	322.7	83.4%	111.4%
11	1	1000	3	67.1	25.3	313.5	78.2%	108.2%
12	1	1000	3	70.0	27.0	300.8	83.6%	103.8%
13	1	1000	6	107.3	35.90	336.1	110.9%	116.0%
14	1	1000	6	114.5	33.6	309.1	104.0%	106.7%
15	1	1000	6	109.5	34.6	298.5	107.0%	103.0%
16	1	1000	9	112.4	31.5	352.9	97.3%	121.8%
17	1	1000	9	115.0	32.7	340.1	101.0%	117.3%
18	1	1000	9	111.3	33.7	363.1	104.1%	125.3%
19	1	1400	3	33.9	36.1	299.4	111.7%	103.3%
20	1	1400	3	37.5	38.7	314.2	119.6%	108.4%
21	1	1400	3	41.7	34.3	301.7	106.0%	104.1%
22	1	1400	6	111.9	34.1	349.1	105.4%	120.5%
23	1	1400	6	112.0	34.5	339.5	106.8%	117.1%
24	1	1400	6	123.8	32.5	364.5	100.6%	125.8%
25	1	1400	9	142.3	30.9	393.8	95.8%	135.9%
26	1	1400	9	136.3	29.0	381.7	89.8%	131.7%
27	1	1400	9	124.2	32.2	397.8	99.6%	137.3%
28	2	600	3	71.2	27.0	339.7	83.6%	117.2%
29	2	600	3	69.3	25.9	331.4	80.0%	114.3%
30	2	600	3	66.3	26.1	320.8	80.6%	110.7%
31	2	600	6	99.4	27.0	347.3	83.5%	119.8%
32	2	600	6	102.2	24.7	319.5	76.4%	110.2%
33	2	600	6	103.9	26.7	321.3	82.6%	110.9%
34	2	600	9	112.9	25.0	342.8	77.5%	118.3%
35	2	600	9	116.4	26.1	335.5	80.7%	115.8%
36	2	600	9	109.1	23.6	326.6	73.0%	112.7%
37	2	1000	3	73.1	35.3	323.9	109.1%	111.8%
38	2	1000	3	69.2	33.7	333.1	104.3%	114.9%
39	2	1000	3	75.9	37.5	340.4	116.0%	117.5%
40	2	1000	6	117.7	35.5	349.3	109.8%	120.5%
41	2	1000	6	110.3	36.5	333.7	112.7%	115.1%
42	2	1000	6	122.5	35.6	330.6	110.1%	114.1%
43	2	1000	9	122.9	29.9	348.7	92.7%	120.3%
44	2	1000	9	136.3	31.2	374.7	96.5%	129.3%
45	2	1000	9	127.6	29.1	374.2	90.0%	129.1%
46	2	1400	3	57.5	33.7	329.1	104.3%	113.6%
47	2	1400	3	74.2	34.2	327.7	105.8%	113.1%
48	2	1400	3	66.3	35.1	341.1	108.7%	117.7%
49	2	1400	6	122.2	28.8	318.9	89.2%	110.0%
50	2	1400	6	119.2	31.1	338.7	96.3%	116.9%
51	2	1400	6	127.5	29.1	326.5	89.9%	112.7%
52	2	1400	9	127.1	24.4	348.6	75.6%	120.3%
53	2	1400	9	120.0	26.9	350.8	83.5%	121.0%
54	2	1400	9	118.1	26.7	363.6	82.8%	125.5%
Min					9.7	194.6		
Max					38.7	397.8		
Mean					28.9	327.6		

^1^ sB reference (unwelded): 32.3 MPa. ^2^ E reference (unwelded): 289.8 MPa.

**Table 3:** Analysis of Variance—ANOVA: E versus Tool; RS; TS.

Source	DoF	SoS	MS	F-Value	*p*-Value
Regression	8	61,908.8	7738.6	28.52	0.000
Tool	1	22,037.4	22,037.4	81.20	0.000
RS	1	7083.4	7083.4	26.10	0.000
TS	1	1419.2	1419.2	5.23	0.027
RS×RS	1	1864.9	1864.9	6.87	0.012
TS×TS	1	183.2	183.2	0.68	0.416
Tool×RS	1	10,357.1	10,357.1	38.16	0.000
Tool×TS	1	7279.6	7279.6	26.82	0.000
RS×TS	1	0.5	0.5	0.00	0.965
Error	45	12,212.2	271.4		
Lack-of-Fit	9	6171.6	685.7	4.09	0.001
Pure Error	36	6040.6	167.8		
Total	53	74,121.0			
R-sq					83.52%
R-sq (adj)					80.59%
R-sq (pred)					76.17%

DoF: Degrees of Freedom. SoS: Sum of Squares. Mean Squares. F: F-value in statistics (also known as Fisher value). *p*: *p*-value in statistics [53].

**Table 4:** Analysis of Variance—ANOVA: sB versus Tool; RS; TS.

Source	DoF	SoS	MS	F-Value	*p*-Value
Regression	8	1963.18	245.398	61.28	0.000
Tool	1	490.16	490.158	122.41	0.000
RS	1	845.98	845.976	211.27	0.000
TS	1	285.78	285.779	71.37	0.000
RS×RS	1	362.64	362.640	90.56	0.000
TS×TS	1	63.62	63.621	15.89	0.000
Tool×RS	1	256.62	256.623	64.09	0.000
Tool×TS	1	181.90	181.902	45.43	0.000
RS×TS	1	212.79	212.789	53.14	0.000
Error	45	180.19	4.004		
Lack-of-Fit	9	119.99	13.333	7.97	0.000
Pure Error	36	60.20	1.672		
Total	53	2143.38			
R-sq					91.59%
R-sq (adj)					90.10%
R-sq (pred)					87.69%

**Table 5:** Analysis of Variance—ANOVA: WT versus Tool; RS; TS.

Source	DoF	SoS	MS	F-Value	*p*-Value
Regression	8	54,643.5	6830.43	70.02	0.000
Tool	1	4920.6	4920.65	50.44	0.000
RS	1	4737.4	4737.38	48.56	0.000
TS	1	3388.4	3388.39	34.73	0.000
RS×RS	1	3403.7	3403.70	34.89	0.000
TS×TS	1	4351.0	4351.02	44.60	0.000
Tool×RS	1	3875.1	3875.06	39.72	0.000
Tool×TS	1	27.6	27.56	0.28	0.598
RS×TS	1	2595.8	2595.84	26.61	0.000
Error	45	4390.0	97.56		
Lack-of-Fit	9	3581.5	397.94	17.72	0.000
Pure Error	36	808.5	22.46		
Total	53	59,033.5			
R-sq					92.56%
R-sq (adj)					91.24%
R-sq (pred)					89.05%

## Data Availability

The data presented in this study are available upon request from the corresponding author.
